# A Survey on the Current Status of Ophthalmological Consultations in Patients With Diabetes Undergoing Maintenance Hemodialysis and the Effectiveness of Education on Consultation Behavior –Experience of a Single Hemodialysis Clinic in Japan

**DOI:** 10.3389/fcdhc.2021.827718

**Published:** 2022-01-26

**Authors:** Moritsugu Kimura, Masao Toyoda, Nobumichi Saito, Makiko Abe, Eri Kato, Akemi Sugihara, Naoto Ishida, Masafumi Fukagawa

**Affiliations:** ^1^ Seichi Clinic, Isehara, Japan; ^2^ Division of Nephrology and Metabolism, Department of Internal Medicine, Tokai University School of Medicine, Isehara, Japan

**Keywords:** ophthalmological consultation, diabetic patient, hemodialysis, education, patient involvement, self-care

## Abstract

**Introduction:**

It is extremely important for patients with diabetes undergoing maintenance hemodialysis (MHD) to receive regular ophthalmologic examinations. However, even in the field of MHD in Japan, where there are many hemodialysis patients and the survival rate is said to be one of the highest in the world, we often see patients with diabetes who do not receive regular ophthalmologic examinations. In this study, we surveyed the status of ophthalmology consultations and the use of diabetic eye notebook (DEN) among hemodialysis patients with diabetes at hemodialysis clinics to confirm the current situation, with the aim of confirming the effectiveness of education on consultation behavior by medical care staff.

**Materials and Methods:**

This study included 38 diabetic hemodialysis patients attending one MHD clinic in Japan for one year from March 2018 to March 2019. In the first fact-finding survey in March 2018, hemodialysis care unit nurses (HCUNs) in the hemodialysis unit asked the diabetic hemodialysis patients whether they had consulted an ophthalmologist and used the DEN. Based on the results, the HCUNs recommended that hemodialysis patients with complications of diabetes be educated about the usefulness of regular ophthalmologic examinations, even during MHD, and that they use the DEN. This was followed by a second fact-finding survey in March 2019 to reconfirm ophthalmology consultations and DEN use.

**Results:**

Regarding the presence of ophthalmology consultations, 22 of 38 (58%) patients had regular ophthalmology consultations in March 2018, and 27 of 38 (71%) patients had consultations in the following year after receiving information from an HCUN. Only 1 of 22 patients (5%) who consulted the ophthalmologist in March 2018 used a DEN, but 19 of 27 patients (70%) used it the following year.

**Conclusion:**

In the future, the development and utilization of a new DEN that includes more detailed patient information, and the spread of self-care guidance to patients by multidisciplinary health care professionals, will increase the consultation rate of MHD patients in Japan and reduce the incidence and progression of ocular diseases in MHD patients.

## Introduction

End-stage renal disease (ESRD) has become an emerging health problem worldwide. The eye shares striking developmental, structural and genetic pathways with the kidney, suggesting that kidney disease and ocular disease may be closely related (1).

In particular, retinopathy affects the progression of nephropathy in patients with diabetic nephropathy, and it is said that early detection and management of patients with retinopathy is important to reduce the risk of death in patients with diabetic nephropathy (2–4). Patients with ESRD are at risk for developing ocular disease. This risk is associated with comorbidities that are common in ESRD patients, as well as to the unique effects of hemodialysis and the uremic state, which can lead to changes in the conjunctivae, cornea, retina, and macula. The most common ophthalmological complaints in ESRD patients include redness, irritation of the eyes, which may be associated with elevation of the product of the serum calcium and phosphorus concentrations, the so-called calcium-phosphorus product or Ca × P. In patients with chronically elevated calcium-phosphate products, band keratopathy may result. Other ophthalmological symptoms include retinal hemorrhage, ischemic optic neuropathy, ophthalmological infection, elevated intraocular pressure, retinal detachment, and macular edema. Prompt recognition that these conditions may threaten a patient’s vision is required (5, 6).

**Figure 1:**
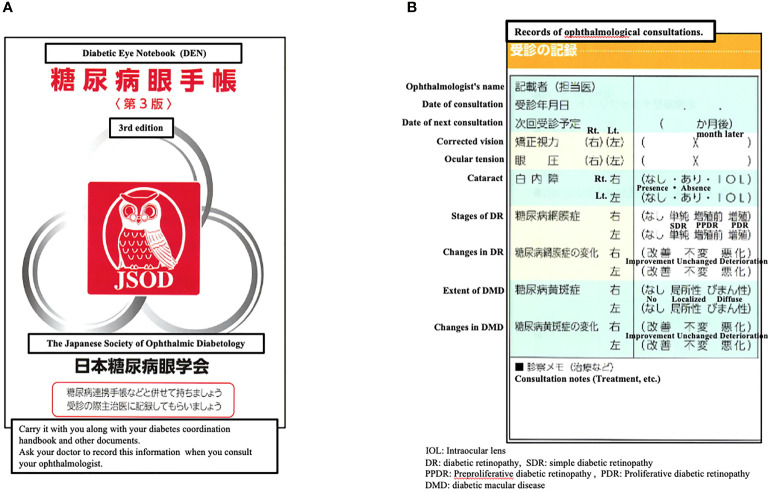
**(A)** The cover of the diabetic eye notebook (DEN). **(B)** The detailed contents of the diabetic eye notebook (DEN).

Moreover, ESRD patients with diabetes mellitus undergoing hemodialysis have a higher incidence of ocular diseases, including diabetic retinopathy (DR), exudative retinal detachment, which can cause a major cause of decreasing visual acuity and blindness (7–11). Therefore, it is very important for patients with diabetes undergoing MHD to have regular ophthalmologic examinations. However, even in the field of MHD in Japan, which has a large number of hemodialysis patients and where the survival rate is said to be among of the highest in the world (12), we often see patients with diabetes who do not receive regular ophthalmologic examinations (12, 13). In addition, collaboration between physicians and ophthalmologists is said to be important in the treatment of diabetes in Japan. **Figures 1A, B** shows a diabetic eye notebook (DEN), the DEN was published by the Japanese Society of Ophthalmic Diabetology in 2002 as one of the solutions to this problem, patients are recommended to use this diary to consult ophthalmologist regularly (14). This study aimed to reduce the incidence and progression of ocular diseases in MHD patients by improving the ophthalmology consultation rate of MHD patients with diabetes, and we surveyed the status of ophthalmology consultations and the use of the DEN among patients with diabetes undergoing MHD at single hemodialysis clinic in order to confirm the current situation and discuss the need for medical collaboration between hemodialysis clinics and ophthalmology clinics and the educational activities for medical staff.

## Materials and Methods

The subjects were 38 patients (males: n=25, females: n=13) among 46 diabetic MHD patients who attended the Sechi Clinic (Isehara City, Kanagawa Prefecture) in March 2018, who continued to attend the clinic until March 2019, excluding patients who were transferred or died. In the first fact-finding survey in March 2018, hemodialysis care unit nurses (HCUNs) asked patients with diabetes undergoing MHD whether they were consulting the ophthalmological clinic and using a DEN. Based on the results, HCUNs verbally explained the three major complications of diabetes to MHD patients with diabetes, educated them on the usefulness of regular ophthalmologic consultations even during MHD because of the risk of blindness, and recommended the use of DEN. The third edition of the DEN was used, and the ophthalmologist’s name, date of consultation, date of next consultation, corrected vision, etc. were recorded by the ophthalmologist. In addition, if there were any diseases, the status, changes, and treatment details were also recorded in “consultation notes” (**Figure 1B**). In the second fact-finding survey in March 2019, as in the first survey, the HCUNs asked MHD patients if they were consulting ophthalmologists and if they were using their DEN. From the medical records of 38 subjects, we also confirmed the history of ophthalmological consultation and the diagnosis of ocular diseases when they were referred to Seichi Clinic for MHD. This is a descriptive study of MHD patients who were followed up for one year after HCUNs awareness campaign.

## Results

**Figure 2:**
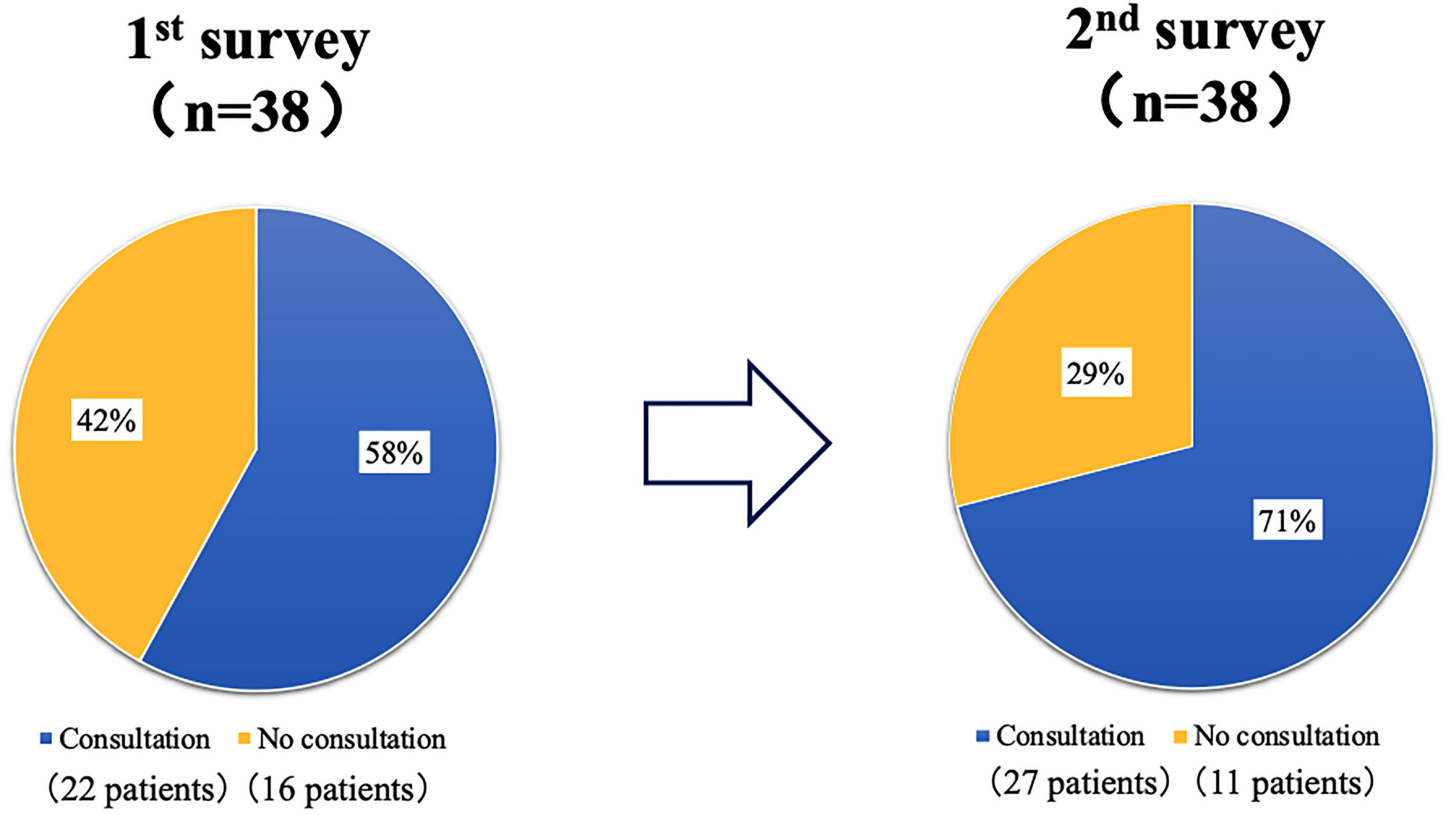
The changes in the presence of ophthalmology consultations before and after the awareness campaign. Regarding the presence of ophthalmology consultations, 22 of 38 (58%) patients had regular ophthalmology consultations in March 2018 (1st survey: the first fact-finding survey in March 2018), and 27 of 38 (71%) patients had consultations in the following year after the awareness campaign (2nd survey: the second fact-finding survey in March 2019).

**Figure 3:**
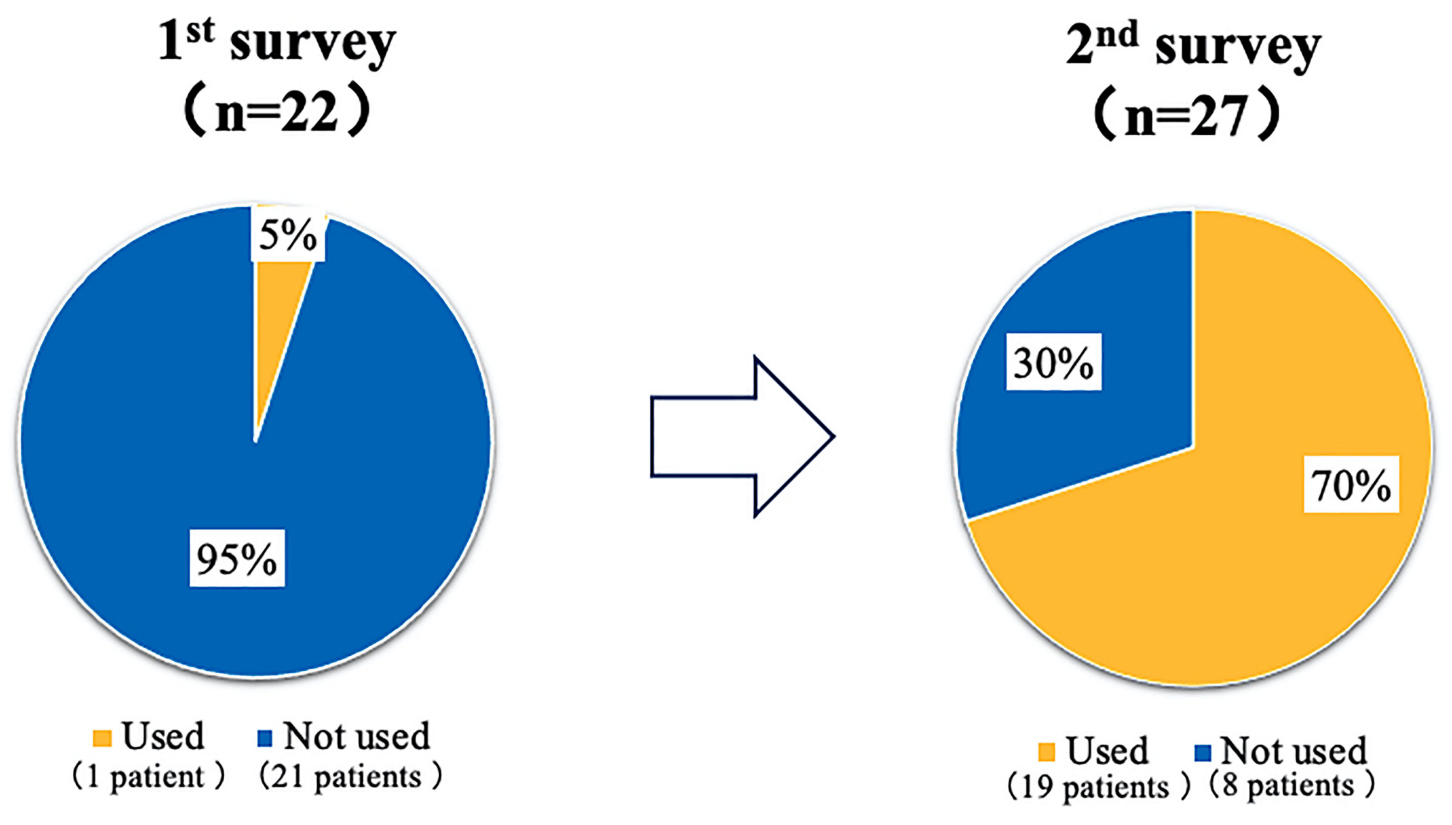
The changes in the presence of the diabetic eye notebook (DEN) use before and after the awareness campaign. Only 1 of 22 patients (5%) who consulted the ophthalmologist in March 2018 (1st survey: the first fact-finding survey in March 2018), used the diabetic eye notebook (DEN), but 19 of 27 patients (70%) used it the following year (2nd survey: the second fact-finding survey in March 2019).

The mean age of the 38 patients was 68.7 years and the mean duration of hemodialysis was 7.2 years. Diabetes mellitus was treated with diet alone in 6 cases. The others received insulin therapy or GLP-1 receptor agonist therapy, or some oral hypoglycemic agents (**Table 1**). Regarding the diagnoses of ocular disease listed on the referral letters of the 38 patients who started MHD at the Seichi Clinic, 1 patient had no ocular disease, in 5 patients, the ocular disease status was unknown, and 32 patients had some kind of ocular disease. Of the 32 patients with confirmed ocular disease, 11 did not consult an ophthalmologist. The most common ocular diseases were DR in 19 patients, cataract in 17 patients, others in 5 patients (**Table 2**). Regarding the presence of ophthalmology consultations, 22 of 38 (58%) patients had regular ophthalmology consultations in March 2018, and 27 out of 38 (71%) patients had consultations in the following year after the awareness campaign (**Figure 2**). Only 1 of 22 patients (5%) who consulted the ophthalmologist in March 2018 used the DEN, but 19 of 27 patients (70%) used it the following year (**Figure 3**).

**Table 1:** Clinical parameters of 38 patients.

Patient Background	
Age (years)	68.7 ± 12.0
Sex (M/F)	25/13
Duration of dialysis (years)	7.2 ± 3.5
Hb (g/dL)	10.0 ± 0.7
Ht(%)	31.7 ± 2.4
HbA1c(%)	5.9 ± 0.9
GA(%)	19.7 ± 6.9
Insulin/GLP-1 receptor agonist (patients)	12/7
Oral medication DPP-4 inhibitor/glinide/α-GI (patients)	11/6/3
Diet therapy only (patients)	6

**Table 2:** Presence or absence of ophthalmological examination, use of DEN and the diagnoses of ocular disease listed on the referral letters of the 38 patients who started MHD.

No	Ocular disease	Ophthalmological examination	Using DEN	No	Ocular disease	Ophthalmological examination	Using DEN
1	PDR	○	×	20	DR	○	×
2	Cataract	×	×	21	DR ▪ Cataract	○	×
3	None	○	×	22	Unknown	×	×
4	PPDR	×	×	23	PDR ▪ Cataract ▪ Other	○	×
5	SDR	○	×	24	DR	○	×
6	PPDR	○	×	25	Unknown	×	×
7	DR	○	×	26	Cataract	○	×
8	PDR ▪ Cataract ▪ Other	○	○	27	Cataract	×	×
9	PDR ▪ Other	○	×	28	Cataract	○	×
10	DR ▪ Cataract	×	×	29	Cataract	×	×
11	DR ▪ Cataract	○	×	30	DR	×	×
12	Unknown	×	×	31	Other	×	×
13	Cataract	○	×	32	Cataract	×	×
14	Cataract	○	×	33	Cataract	○	×
15	PDR	○	×	34	Cataract	×	×
16	DR	○	×	35	DR	○	×
17	Other	×	×	36	Unknown	×	×
18	Unknown	×	×	37	DR	×	×
19	Cataract	○	×	38	DR ▪ Cataract	○	×

DR, diabetic retinopathy; SDR, simple diabetic retinopathy, PPDR, preproliferative diabetic retinopathy; PDR, proliferative diabetic retinopathy; DEN, diabetic eye notebook.

○: The patient had an ophthalmologicalexamination.The patient used DEN.

×: The patient did not have an ophthalmological examination. The patient did not use DEN.

## Discussion

In this study, HCUNs explained the necessity of ophthalmologic examinations to patients at a MHD clinic, which resulted in a 10% increase in the ophthalmological examination rate from approximately 60% to approximately 70% after one year, and the utilization rate of DEN increased significantly from 5% to 70%. The reason why the HCUNs were able to achieve these results after only one year of educational activities may be that the effects of the collaboration between the physician, HCUNs and ophthalmologist before the introduction of MHD remained. However, considering the fact that 30% of patients had still not consulted ophthalmological clinics and that 30% of patients who did consult a clinic did not use the DEN. This may be due to the following problems: 1) dialysis physicians do not cooperate with ophthalmologists or physicians before the introduction of hemodialysis, and 2) self-care education for hemodialysis patients is not as complete as it was before the introduction of dialysis.

### 1) Dialysis Physicians Do Not Cooperate With Ophthalmologists or Physicians Before the Introduction of Hemodialysis

The fact that 11 of the 32 patients who had consulted an ophthalmologist before starting hemodialysis and who had been diagnosed with some type of ocular disease did not consult an ophthalmologist (**Table 2**) suggests that the physician and ophthalmologist may have collaborated prior to the start of dialysis, but that the dialysis physician may have stopped working with the ophthalmologist once dialysis started. In Japan, the importance of collaboration between physicians and ophthalmologists from the early stages of diabetes has been reported for some time (15, 16), and in 2002, the Japanese Diabetic Eye Society issued the DEN, which recommends regular consults to ophthalmologists, as a means of collaboration (14). On the other hand, there are no reports on collaboration between dialysis doctors and ophthalmologists. However, considering the fact that dialysis patients are at high risk for the development of various ocular diseases (5, 6), it is important for dialysis physicians along with HCUNs to collaborate more closely with ophthalmologists using collaboration tools such as the DEN, and more case reports are expected. In addition, while the DEN is an effective handbook for collaboration with ophthalmologists, it does not have a column for important patient information such as weight, blood glucose, lipid profile, or liver/renal function, etc., even though there is a column for HbA1c levels. This information can only be provided in the “Consultation notes” section (**Figure 1B**). In addition, in the current 4th edition, the “Consultation notes” section has been deleted, making it difficult to describe additional information. In order for ophthalmologists, physicians, dialysis physicians and HCUNs to better collaborate on patient information in the future, it may be necessary to develop a new DEN that can describe this information.

In other countries, it is said that a partnership between primary care physicians and ophthalmologists is the only way to save many people at risk of diabetic retinopathy. Furthermore, in Japan, when diabetic nephropathy transitions to end-stage renal failure, the doctor in charge is often changed from a diabetologist to a nephrologist or a dialysis specialist (15). Under these circumstances, the use of a handbook such as DEN as a tool for understanding the clinical course of eye disease and for continuing cooperation with ophthalmologists is expected to help maintain the quality of life of many diabetic dialysis patients in Japan and abroad.

### 2) Self-Care Education for Hemodialysis Patients Is Not as Complete as It Was Before the Introduction of Hemodialysis. Importance of Education and Team Care for Patients With Diabetes Undergoing MHD

Japan has very few kidney transplants and many MHD patients in comparison to the prevalence of ESRD (12). In addition, the survival rate of MHD patients is one of the highest in the world (13). Japanese clinical practice patterns differ from those of other countries in many ways, including the reasons for the longevity of MHD patients, as reported in the DOPPS (Dialysis Outcomes and Practice Patterns Study) study (17, 18).

This is reflected by the fact that the rate of ophthalmologic examinations and the use of DEN significantly increased only one year after the initiation of the awareness campaign by the HCUNs; however, the results of the first survey confirmed the fact that there has been a lack of awareness-raising activities and patient self-care education for ophthalmologic examinations. In recent years, “patient involvement in healthcare” has been attracting international attention as a way to achieve safe, high-quality healthcare. Patient involvement in healthcare means that patients and their families collaborate with medical professionals to improve the quality and safety of medical care, and the modes of participation are said to include a wide range of areas, not only at the level of the individual patient (e.g., decisions about treatment choices and self-care) but also in relation to hospital management and other areas (19–22). In this focus on the importance of patient involvement in healthcare, the importance of self-care education for diabetes has long been reported in Japan (23, 24), and steps have been taken to allow reimbursement for patient education and guidance to prevent dialysis. However, the importance of self-care education after the initiation of hemodialysis has not been emphasized as much. Through this survey, it was confirmed patients with diabetes require thorough self-care education before and after the initiation of hemodialysis in order to enhance patient involvement in healthcare. The hemodialysis unit team in Japan is usually composed of several healthcare professionals (e.g., nurses, clinical engineers, dieticians, and doctors) (25). If all of these medical professionals involved in dialysis understand the existence of DEN and can provide the same self-care guidance to patients as they did before the introduction of dialysis, it is estimated that more MHD patients will consult ophthalmological clinics.

## Conclusion

This study shows that the rate of ophthalmological consultation and the rate of DEN use can be increased by the awareness campaign by HCUNs. In the future, the development and utilization of a new DEN that includes more detailed patient information, and the spread of self-care guidance to patients by multidisciplinary health care professionals, will increase the consultation rate of MHD patients in Japan and reduce the incidence and progression of ocular diseases in MHD patients.

It is difficult to demonstrate the effectiveness of these results in countries with different healthcare systems from Japan. Longer-term studies involving other countries and other facilities are needed. However, the findings suggest that it is not detrimental for patients with diabetes in any country to have healthcare providers who are involved in their care to encourage and educate patients to seek ophthalmological care, and to use services such as the DEN. Based on the results of this study, we hope that more medical professionals around the world will become aware of patient involvement in healthcare and educate patients about ophthalmologic examinations for diabetic hemodialysis patients using tools such as the DEN, so that the quality of life of patients can be maintained and improved as much as possible.

## Data Availability Statement

The original contributions presented in the study are included in the article/supplementary material. Further inquiries can be directed to the corresponding author.

## Ethics Statement

The studies involving human participants were reviewed and approved by Toyu Medical Research Ethics Review Committee. Written informed consent for participation was not required for this study in accordance with the national legislation and the institutional requirements. Informed consent was received from patients for the submissionof this manuscript to an academic journal.

## Author Contributions

MK, MT, AS, NI, and MF contributed to conception and design of the study. NS, MA, and EK organized the database. MK, MT, and NS performed the statistical analysis. MK and MT wrote the manuscript. All authors contributed to manuscript revision, read, and approved the submitted version.

## Conflict of Interest

The authors declare that the research was conducted in the absence of any commercial or financial relationships that could be construed as a potential conflict of interest.

## Publisher’s Note

All claims expressed in this article are solely those of the authors and do not necessarily represent those of their affiliated organizations, or those of the publisher, the editors and the reviewers. Any product that may be evaluated in this article, or claim that may be made by its manufacturer, is not guaranteed or endorsed by the publisher.
